# Monitoring regulation of DNA repair activities of cultured cells in-gel using the comet assay

**DOI:** 10.3389/fgene.2014.00232

**Published:** 2014-07-16

**Authors:** Catherine M. Nickson, Jason L. Parsons

**Affiliations:** Department of Molecular and Clinical Cancer Medicine, North West Cancer Research Centre, University of LiverpoolLiverpool, UK

**Keywords:** base excision repair, DNA repair, DNA damage, ubiquitin, comet assay, ubiquitylation, DNA polymerase β, polynucleotide kinase phosphatase

## Abstract

Base excision repair (BER) is the predominant cellular mechanism by which human cells repair DNA base damage, sites of base loss, and DNA single strand breaks of various complexity, that are generated in their thousands in every human cell per day as a consequence of cellular metabolism and exogenous agents, including ionizing radiation. Over the last three decades the comet assay has been employed in scientific research to examine the cellular response to these types of DNA damage in cultured cells, therefore revealing the efficiency and capacity of BER. We have recently pioneered new research demonstrating an important role for post-translational modifications (particularly ubiquitylation) in the regulation of cellular levels of BER proteins, and that subtle changes (∼20–50%) in protein levels following siRNA knockdown of E3 ubiquitin ligases or deubiquitylation enzymes can manifest in significant changes in DNA repair capacity monitored using the comet assay. For example, we have shown that the E3 ubiquitin ligase Mule, the tumor suppressor protein ARF, and the deubiquitylation enzyme USP47 modulate DNA repair by controlling cellular levels of DNA polymerase β, and also that polynucleotide kinase phosphatase levels are controlled by ATM-dependant phosphorylation and Cul4A–DDB1–STRAP-dependent ubiquitylation. In these studies we employed a modification of the comet assay whereby cultured cells, following DNA damage treatment, are embedded in agarose and allowed to repair in-gel prior to lysis and electrophoresis. Whilst this method does have its limitations, it avoids the extensive cell culture-based processing associated with the traditional approach using attached cells and also allows for the examination of much more precise DNA repair kinetics. In this review we will describe, using this modified comet assay, our accumulating evidence that ubiquitylation-dependant regulation of BER proteins has important consequences for overall cellular DNA repair capacity.

## THE BASE EXCISION REPAIR (BER) PATHWAY

The human genome is constantly exposed to agents that cause damage to DNA, for example endogenously through products of cellular oxidative metabolism, and exogenously through environmental agents such as ionizing radiation. These agents can cause damage to the DNA phosphodiester backbone resulting in the formation of DNA strand breaks, attack to DNA bases resulting in oxidation events (e.g., 8-oxoguanine) or even cause loss of the DNA base itself (apurinic/apyrimidinic or AP site). Such events have been estimated to occur at approximately 10,000 per human cell per day (Lindahl, 1993), and if left unrepaired, these types of DNA damage have been implicated in the development of several human disorders, such as in premature aging, in neurodegenerative diseases, and in cancer. Consequently, the base excision repair (BER) pathway has evolved as the major cellular system which is directly involved in the removal and repair of damaged DNA bases, as well as DNA single strand breaks (SSB) that contain various modifications on the 5′- and/or 3′-end (Parsons and Dianov, 2013). BER is therefore a vital DNA repair pathway directly involved in the maintenance of genome stability and consequently contributes to suppressing the development of human diseases.

**FIGURE 1:**
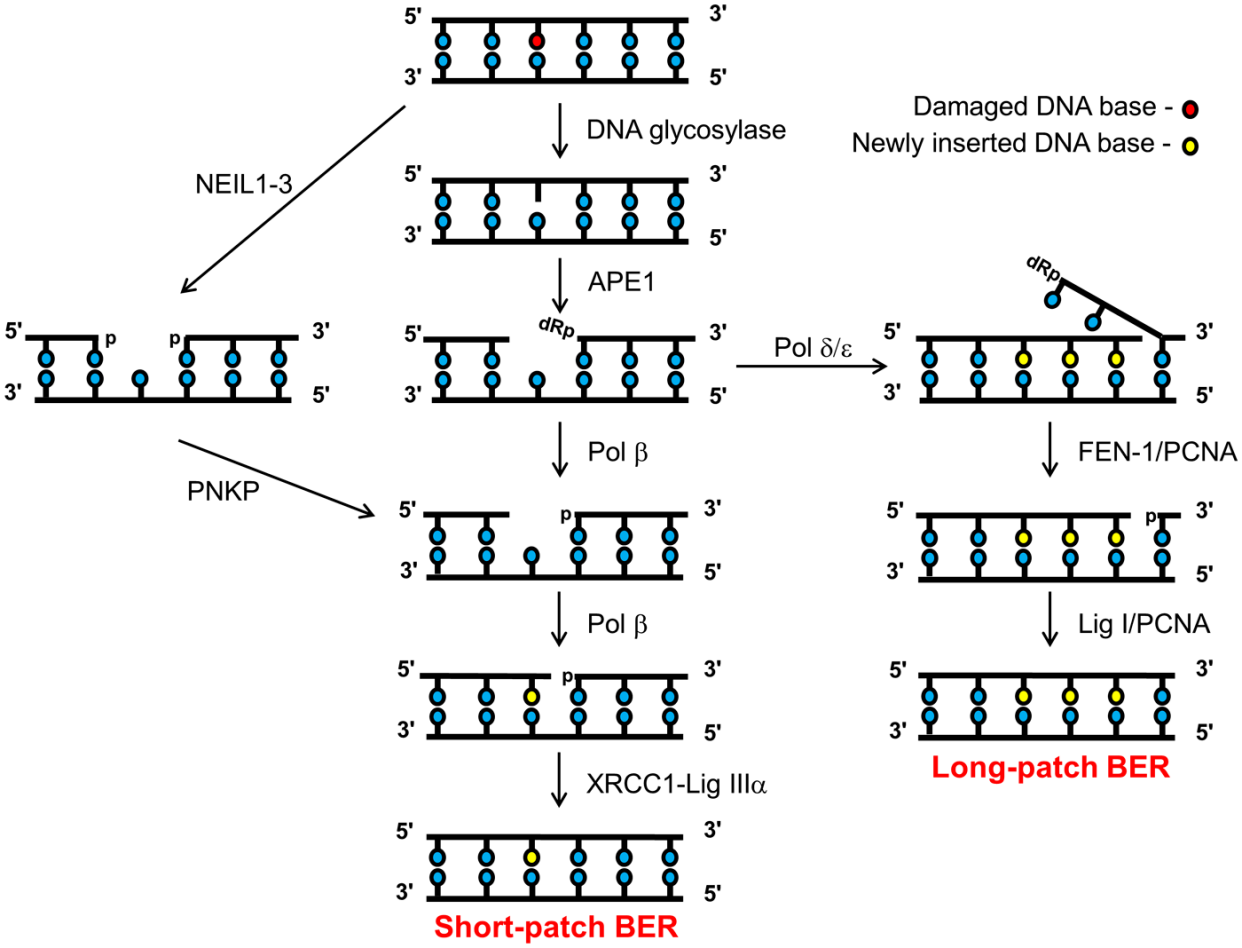
**Schematic of the BER pathway.** Base excision repair (BER) is initiated by a damage specific DNA glycosylase that excises the damaged base to create an abasic site, which is then incised by APE1 creating a DNA SSB flanked by 3′-hydroxyl and 5′-dRP ends. Pol β cleaves the 5′-dRP moiety and simultaneously adds a single correct nucleotide into the one-nucleotide gap. Finally, the DNA SSB ends are sealed by the XRCC1-Lig IIIα complex, which completes the short-patch BER pathway (central branch). However, if the 5′-dRP moiety is resistant to cleavage by Pol β, then a polymerase switch occurs involving the recruitment of Pol δ/ε which add 2–8 more of the correct nucleotides into the repair gap. Pol δ/ε activity creates a 5′-flap structure that is subsequently excised by FEN-1 in a PCNA-dependent manner. The remaining DNA SSB ends are then sealed by Lig I to complete the long-patch BER pathway (right branch). Alternatively, repair of oxidized DNA bases initiated by the NEIL (1–3) glycosylases, generates a one nucleotide gap flanked by 3′- and 5′-phosphate ends (left branch). The 3’-phosphate is removed by PNKP, generating a substrate amenable for one nucleotide insertion by Pol β and subsequent ligation by XRCC1-Lig IIIα complex.

BER can be divided into several major steps, each of which is performed by a specific enzyme or class of enzymes that recognize and process the DNA damage or DNA damage intermediate. The majority of BER is achieved through the short-patch pathway that involves the removal and replacement of a single damaged base (Dianov et al., 1992; **Figure 1**, central scheme). In the first step, a damage-specific DNA glycosylase excises the damaged base by cleavage of the N-glycosylic bond linking the damaged base to the sugar phosphate backbone. Currently, there are eleven known human DNA glycosylases, each has its own substrate specificity (Jacobs and Schar, 2012). For example, 8-oxoguanine DNA glycosylase (OGG1) is the major DNA glycosylase involved in the excision of 8-oxoguanine residues, whereas endonuclease III homolog (NTH1) excises oxidized pyrimidines, such as thymine glycol, 5-hydroxycytosine, and 5-hydroxyuracil. Once the damaged DNA base is removed, the second step is performed by AP endonuclease-1 (APE1) which incises the phosphodiester- backbone 5′- to the abasic site to create a DNA SSB flanked by 3′-hydroxyl and a 5′-deoxyribosephosphate (dRP) ends (Demple et al., 1991; Robson and Hickson, 1991). Step three involves removal of the 5′-dRP end carried out by the dRP lyase activity of DNA polymerase β (Pol β), which also simultaneously inserts the complementary nucleotide into the DNA repair gap thus generated (Dianov et al., 1992; Matsumoto and Kim, 1995; Sobol et al., 1996). In the final step DNA ligase IIIα, which is in a complex with X-ray cross complementing protein-1 (XRCC1), then seals the remaining nick in the DNA backbone to restore genome integrity (Cappelli et al., 1997; Nash et al., 1997). The pathway described above is employed, in the main, for the repair of >80% of damaged DNA bases and is commonly referred to as the short-patch BER pathway (Dianov and Parsons, 2007). In instances (within step 3) where the 5′-end is resistant to the dRP lyase activity of Pol β, then there is a polymerase switch to the replicative DNA polymerases, Pol δ/ε. These DNA polymerases add several (2–8) nucleotides into the repair gap, thus creating a 5′-flap structure (**Figure 1**, right scheme). This flap structure is recognized and excised by flap endonuclease-1 (FEN-1), in association with the processivity factor proliferating cell nuclear antigen (PCNA). Finally DNA ligase I (Lig I) then seals the remaining nick in the DNA backbone to complete the long patch BER pathway (Frosina et al., 1996; Podlutsky et al., 2001).

Over the last 10 years, a further sub-pathway of BER has been uncovered through the discovery of the endonuclease VIII-like (NEIL) DNA glycosylases (**Figure 1**, left scheme). Rather than generating an AP site for APE1 activity, the enzymes (NEIL1, 2, and 3) excise the damaged base creating a DNA SSB flanked with 5′- and 3′-phosphate ends (Hazra et al., 2002a,b; Takao et al., 2002; Liu et al., 2010). The 3′-phosphate subsequently requires removal by polynucleotide kinase phosphatase (PNKP), which then creates the 3′-hydroxyl end that is required for Pol β activity (Wiederhold et al., 2004). Following nucleotide insertion, the nick is finally sealed by DNA ligase IIIα-XRCC1, as per the short-patch BER pathway. The NEIL glycosylases appear to have a similar substrate specificity to the major oxidative DNA glycosylases, OGG1 and NTH1, in that they recognize oxidized purines and pyrimidines. However, it is currently unknown what proportion of these oxidized DNA bases are repaired through the NEIL-dependant pathway. Intriguingly, there is some suggestion that these enzymes have a preference for specific, novel oxidative DNA damage, or that they have a preference for single stranded DNA or DNA bubble structures that may be generated through DNA replication (Dou et al., 2003; Hailer et al., 2005; Parsons et al., 2005, 2007; Chan et al., 2009; Zhou et al., 2013).

## REGULATION OF BER THROUGH THE UBIQUITIN PROTEASOME PATHWAY (UPP)

BER proteins, particularly over the last decade, have been discovered to be subject to post-translational modifications, such as phosphorylation, acetylation, and ubiquitylation, that have been shown to regulate protein activity, cellular localization, protein–protein interactions, as well as protein stability (Almeida and Sobol, 2007). Recently, there has been accumulating interest in ubiquitylation mediated through the ubiquitin proteasome pathway (UPP), as a means of regulating BER proteins, particularly the cellular steady state levels of BER proteins but also those in response to oxidative stress (Dianov et al., 2011; Parsons and Dianov, 2013). This is particularly important since BER misregulation leading to altered enzymes levels has been frequently observed in several human disorders, such as in premature aging, in cancer, and in neurodegenerative diseases (Coppede and Migliore, 2010; Wilson et al., 2011; Wallace et al., 2012). This evidence highlights a critical role for regulating BER capacity in the maintenance of genome stability and in human disease prevention. Interestingly, BER protein levels do not change dramatically in the cellular response to acute DNA damage induced by exogenous mutagens, suggesting that mammalian cells have a limited capacity to be able to repair the ensuing DNA damage. This suggests that DNA damage repair is achieved by multiple repair cycles of available BER enzymes although if the repair capacity of the cell is exceeded for a significant length of time, then the cell may undergo apoptosis thus avoiding the accumulation of genetic alterations. There are now data emerging to suggest that the cellular steady-state levels of BER enzymes, and therefore the corresponding DNA repair capacity, are adjusted to the cellular levels of DNA damage so that the rate of generation of DNA lesions is comparable to the rate of their immediate repair (Parsons et al., 2008). Indeed, the UPP has been discovered to play a vital role in modulating the BER capacity of the cell and adjusting it to the cellular levels of endogenous DNA damage.

**FIGURE 2:**
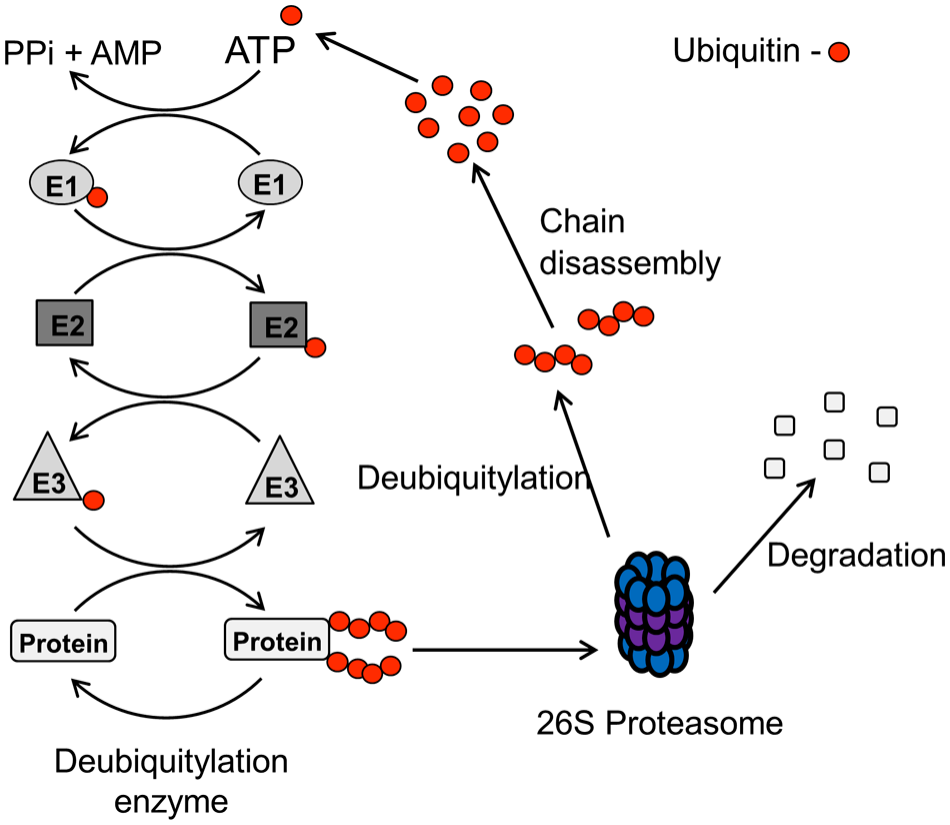
**Schematic of the UPP pathway.** The UPP pathway is initiated by an E1 activating enzyme in an ATP-dependant process which forms a thioester with the ubiquitin molecule. The activated ubiquitin molecule is then transferred to a ubiquitin conjugating enzyme (E2), and the E2-ubiquitin complex then binds with an E3 ubiquitin ligase and the target protein. The E3 ubiquitin ligase transfers the ubiquitin moiety onto specific lysine residues within the target protein, and the formation of ubiquitin chains through internal lysine residues within the ubiquitin protein (termed polyubiquitylation), usually targets the protein for degradation by the 26S proteasome. Protein degradation is achieved by deubiquitylation, and subsequent disassembly, of the ubiquitin chains from the ubiquitylated protein which is then degraded, and the ubiquitin protein is recycled.

The UPP involves adding the ubiquitin moiety (76 amino acids, 8 kDa protein) onto specific lysine residues on the target protein, and is performed by a cascade of enzymes (**Figure 2**; Weissman et al., 2011). The UPP is initiated by an E1 activating enzyme which forms a thioester with the ubiquitin molecule. The activated ubiquitin molecule is then transferred to a ubiquitin conjugating enzyme (E2) that complexes with an E3 ubiquitin ligase and the target protein, which is then modified with the ubiquitin moiety on particular lysine residues. The specificity of the pathway is achieved at the level of the E3 ubiquitin ligases, since these bind and target specific proteins for ubiquitin attachment. Indeed, >500 E3 ubiquitin ligases are thought to exist in human cells and these can be classified into HECT (homologous to E6-associated protein C-terminus), RING (really interesting new gene) and U-Box domain containing enzymes. When a target protein is modified with a single ubiquitin molecule (termed monoubiquitylation), this usually is involved in regulating protein activity, cellular localization or protein–protein interactions. In contrast, the addition of branched ubiquitin chains, which are formed through internal lysines on the ubiquitin molecule (i.e., K6, K11, K27, K29, K33, K48, and K63), also termed polyubiquitylation, usually targets the protein to the proteasome (particularly K48 linkages) where it is subsequently degraded.

In addition to E3 ubiquitin ligases, enzymes that are able to reverse the effects of ubiquitylation exist, which are known as deubiquitylation enzymes. Approximately 90 deubiquitylation enzymes have been shown to be present in human cells that are able to hydrolyse ubiquitin chains, and they consist of five families of enzymes. These are the ubiquitin specific proteases (USPs), ubiquitin COOH-terminal hydrolases (UCHs), ovarian tumor proteases, Josephins, and the JAB1/MPN/MOV34 (JAMMs) family (Clague et al., 2013). Deubiquitylation enzymes also appear to demonstrate a degree of substrate specificity, and therefore play a critical role in the regulation of the levels of key cellular proteins.

Only recently has the UPP been shown to play an important role in BER regulation by modulating cellular levels of key BER proteins (Parsons and Dianov, 2013). Indeed, both monoubiquitylation and polyubiquitylation of BER proteins has been discovered. Monoubiquitylation has been shown to regulate cellular localization and/or protein activity, as well as being a precursor for subsequent polyubiquitylation that usually targets the protein for proteasomal degradation. Consequently, these cellular mechanisms play important roles in the response to acute DNA damage, in which BER proteins levels can marginally increase to accommodate a small increase in DNA damage load. This suggests that the levels of BER proteins are finely tuned to the amount of endogenous DNA damage. There is also emerging evidence suggesting considerable crosstalk between BER protein ubiquitylation and other post-translational modifications, such as phosphorylation, which have been shown to control BER protein levels via modulating ubiquitylation-dependant degradation.

## MEASURING DNA REPAIR IN-GEL USING THE COMET ASSAY

The comet assay, also known as the single cell gel electrophoresis assay, is a very sensitive and rapid quantitative technique used to detect DNA damage at the individual cell level. Originally developed in 1984 by two Swedish scientists, Ostling and Johanson (1984), it has since become widely used as a technique for the evaluation of DNA damage in a wide variety of cell types. The assay allows for visual evidence of DNA damage in a eukaryotic cell to be measured, based on the quantification of DNA containing breaks migrating from the cell nucleus during electrophoresis, to generate the characteristic “comet” images following DNA staining and image analysis. The most widely performed version is the alkaline comet assay (Singh et al., 1988), since the DNA unwinding and electrophoresis steps are performed in high alkaline buffer, which reveals the presence of alkali-labile sites (AP sites), in addition to DNA double strand breaks and DNA SSBs. As well as measuring DNA damage, the assay can also be employed at various time points post-treatment of cells, to measure the rate of DNA damage repair. Indeed, the comet assay is widely used to measure cellular DNA repair capacity, and also to monitor changes in this repair capacity in repair-deficient cells, or in cells that have been genetically manipulated. For example, XRCC1-deficient cells which are unable to perform short-patch BER, have been found to exhibit lower DNA repair rates by the comet assay (Taylor et al., 2000, 2002).

Practically, the assay to measure DNA damage repair involves several steps; treatment of the cells with a DNA damaging agent, a period of incubation time to allow for DNA repair, embedding of the cells in agarose, cell lysis, DNA unwinding, DNA migration by electrophoresis, and finally staining of the DNA for image analysis. Traditionally, a confluent monolayer of cells in tissue culture flasks or dishes is treated with a DNA damaging agent. Following treatment, the cells are washed with buffer (e.g., phosphate buffered saline) to remove the majority of the genotoxin, and then fresh culture medium added to the cells prior to incubation at 37^∘^C in a CO_2_ incubator to allow for DNA repair. The attached cells are then removed from the flasks/dishes by trypsinization involving a further incubation at 37^∘^C. Cells are subsequently counted, diluted to the appropriate cell density and mixed with molten agarose (at ∼35^∘^C) prior to embedding onto a microscope slide using a glass coverslip. The agarose is then allowed to set by placing the slides on ice and the coverslip removed prior to immersion of the slide containing the agarose embedded cells in lysis buffer. In combination with this approach, the use of recombinant DNA repair proteins, such as Fpg and Nth1 that recognize oxidized purine and pyrimidines, respectively, can be used to convert oxidized DNA bases to DNA strand breaks and therefore reveal the true extent of the DNA damage and its subsequent repair (Azqueta and Collins, 2013). Whilst this method has the advantage of allowing all the cells to be exposed to a DNA damaging agent at once, thus eliminating variation in exposure levels, the use of trypsin to detach the cells requires caution. If the amount, and incubation time, with trypsin is not correctly controlled, this can increase the basal level of DNA strand breaks therefore causing variation in the amount of DNA damage quantified. Furthermore, this method is extremely laborious and time-consuming, considering the multiple time points (i.e., >5) that require analysis (involving separate cell culture flasks/dishes that necessitate trypsinization and processing). This approach also does not allow the entirely accurate measurement of DNA repair kinetics post-treatment due to the extended cell manipulations prior to embedding in agarose.

**FIGURE 3:**
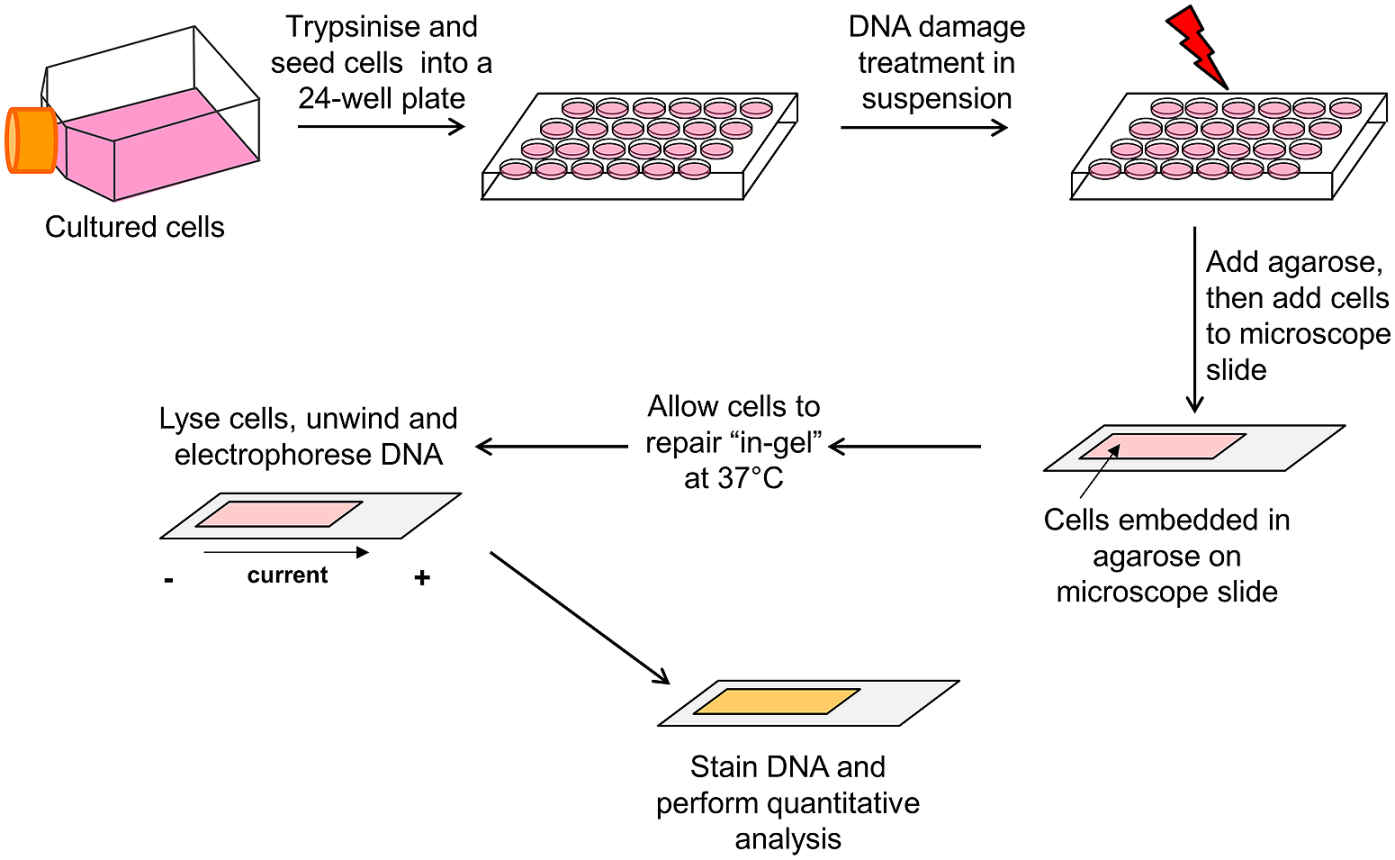
**Schematic of the in-gel DNA repair comet assay.** Actively dividing cells cultured as a monolayer in flasks/dishes are trypsinized, counted, and diluted in cell culture medium (∼2 × 10^5^ cells/ml). Cells (250 μl) are aliquoted into the wells of a 24-well plate placed on ice, and then treated in suspension with the DNA damaging agent. Molten low melting point agarose (1 ml) is added to the cells, mixed, and the agarose/cell suspension immediately added to a microscope slide precoated with normal melting point agarose. A glass coverslip is added and the slide placed on ice to allow the agarose to set. The slides are then transferred to a humidified chamber prewarmed at 37^∘^C and the cells allowed to undergo DNA damage repair for the appropriate times. Following incubation, the glass coverslip is removed and the slides placed in cell lysis buffer for at least an hour at 4^∘^C. Following cell lysis, the slides are transferred to an electrophoresis tank containing DNA unwinding/electrophoresis buffer for 30 min to allow the DNA to unwind, and the DNA electrophoresed (25 V, 300 mA for 25 min) to allow DNA migration. The slides are washed with neutralization buffer, allowed to dry overnight and the following day, the agarose is rehydrated and the DNA stained. The slides are allowed to dry again, prior to subsequent quantitative analysis of DNA strand break and alkali labile site measurement using the appropriate image analysis software.

As an alternative to the traditional approach (treatment of cultured cells as a monolayer with a genotoxin, followed by incubation prior to cell trypsinization) for measuring DNA repair using the comet assay as described above, variations of this method have been described. For example, one study irradiated cells already embedded in agarose, and the slides were consequently placed in medium to allow for DNA repair prior to cell lysis (Alapetite et al., 1999). We have also more recently described another variation of this method by treating cells in a suspension of medium with a genotoxin, embedding the cells within an agarose matrix and then allowing the cells to repair the DNA damage *in situ* in an humidified chamber (Parsons and Elder, 2003; Woodhouse et al., 2008). In our opinion, this method is less laborious and time-consuming, and can allow the measurement of DNA damage at more precise time points post-treatment. We will therefore detail the major steps involved in this in-gel DNA repair alkaline comet assay (**Figure 3**).

Following trypsinization of actively dividing cells, the cells are counted and diluted accordingly in cell culture medium (∼2 × 10^5^ cells/ml). The cells are then aliquoted (250 μl/well) into the wells of a 24-well plate which is placed on ice to prevent cell adhesion. The cells can then be treated in suspension on ice with the DNA damaging agent, and in particular we have previously used either ionizing radiation or hydrogen peroxide, due to its relatively short half-life in solution. Following DNA damage treatment, the cells are mixed with molten (at ∼35^∘^C) low melting point agarose (1 ml of 1% agarose in PBS) and immediately the agarose/cell suspension (1 ml) is removed. The suspension is added to a microscope slide (76 mm × 26 mm), which had already been precoated with normal melting point agarose (1 ml of 1% agarose in water) and allowed to dry. The cell/agarose mix is covered with a glass coverslip (22 mm × 50 mm) and the slide transferred to a metal tray, on ice, to stimulate the agarose to set. After 2–3 min on ice, the slides can then be transferred to a humidified chamber prewarmed at 37^∘^C (slide box containing damp tissue to create a humid environment), and the cells allowed to undergo DNA damage repair for the appropriate times (i.e., 5–120 min). Following incubation, the slides are removed from the humidified chamber, the coverslip removed and the slides placed in cell lysis buffer containing high salt, detergent, and DMSO [10 mM Tris (pH 10.5), 2.5 M NaCl, 100 mM EDTA, plus 1% DMSO, and 1% Triton X-100; prepared just before use] at 4^∘^C. This step will halt the DNA repair reaction as the cell lysis buffer destroys all cellular membranes and constituents, leaving the DNA intact. Cells are subsequently allowed to lyse for at least an hour (overnight is also possible), and then placed in an electrophoresis tank (darkened thus avoiding light exposure and prevent additional DNA damage induction) and covered with DNA unwinding/electrophoresis buffer (300 mM NaOH, 1 mM EDTA, and 1% DMSO prepared just before use) for 30 min to allow the DNA to unwind. The DNA is electrophoresed at 25 V for 25 min (at 300 mA) to allow the DNA to migrate, after which the slides are covered with neutralization buffer (500 mM Tris–HCl, pH 8.0) for 3 × 5 min washes and the agarose then allowed to dry overnight. The following day, the agarose slides are rehydrated in water (pH 8.0) for 30 min prior to staining (we routinely use 1 ml SYBR Gold diluted at 1:10,000 for 30 min). The slides are allowed to dry again, prior to subsequent analysis (i.e., 50 cells per slide and >2 slides per time treatment; analysis performed using Komet 5.5 from Andor Technology, Belfast, UK). The slides can be stored indefinitely in a dry box in the dark, and rehydrated and restained, if necessary. The in-gel DNA repair comet assay described should be repeated in at least three independent experiments, to ensure reproducibility.

It should be noted that whilst this modified comet assay has its advantages (i.e., less laborious and time-consuming, thus avoiding extensive cell culture based-processing, and allowing more precise DNA damage repair kinetics especially at earlier time points), there is the limitation that any DNA damaging agent with a significantly long half-life in solution cannot be used for treating the cells in suspension. This is since the agent will remain in contact with the cells and continue to damage the DNA during the DNA repair time course period.

## MONITORING BER REGULATION USING THE IN-GEL DNA REPAIR COMET ASSAY

We have successfully used the modified alkaline comet assay described above, whereby cells treated in suspension with a DNA damaging agent are embedded in agarose and subsequently allowed to repair in-gel prior to lysis and electrophoresis, to study DNA damage repair kinetics in response to oxidative stress. Specifically, we have most recently employed this technique to strengthen our accumulating evidence that regulation of BER protein levels through the UPP has important consequences for cellular DNA repair capacity, particularly in response to exogenous stress. Principally, we have focussed on the regulation of the cellular levels of the major DNA polymerase employed in BER, Pol β, through the UPP. However, we have also recently examined the cellular mechanism of regulation of PNKP protein levels by the UPP. We will therefore summarize the key important findings of these studies, including how the in-gel DNA repair comet assay has provided vital information on DNA damage repair kinetics through BER protein modulation. We will also briefly summarize key evidence to date, highlighting an important role for the UPP in the regulation of other BER enzymes.

### REGULATION OF POL β PROTEIN LEVELS

Among the BER proteins, Pol β has a very important role in filling the one nucleotide gap that arises during the BER process. The regulation of cellular Pol β protein levels is vital as haploinsufficiency, resulting in reduced BER capacity, has been shown to increase aging and the susceptibility to human diseases, such as cancer (Cabelof et al., 2006; Patterson and Cabelof, 2012). Furthermore, Pol β overexpression has been shown to cause a mutator phenotype in cells (Canitrot et al., 1998) and also Pol β has shown to be overexpressed in approximately 30% of all human cancers (Albertella et al., 2005). Only over the last 5–6 years have we begun to understand the mechanism of regulation of cellular Pol β protein levels, and indeed the crucial role for the UPP in this process. Firstly, in pioneering work, we demonstrated that Pol β is stabilized on damaged DNA in a complex with DNA ligase IIIα-XRCC1 and therefore Pol β protein levels are controlled by the level of endogenous DNA damage (Parsons et al., 2008). In this study we showed that Pol β protein not involved in a complex is targeted for ubiquitylation-dependant proteasomal degradation, as evidenced by decreased Pol β protein levels in XRCC1-deficient cells. The E3 ubiquitin ligase involved in polyubiquitylation of Pol β was discovered using an *in vitro* ubiquitylation assay incorporating Pol β as a substrate in combination with fractionated cell extracts, and was identified as C-terminus of Hsc70 interacting protein (CHIP). The role of CHIP in modulating the steady state levels of Pol β was confirmed as observed by increased Pol β levels in HeLa cells following CHIP siRNA-mediated knockdown.

**FIGURE 4:**
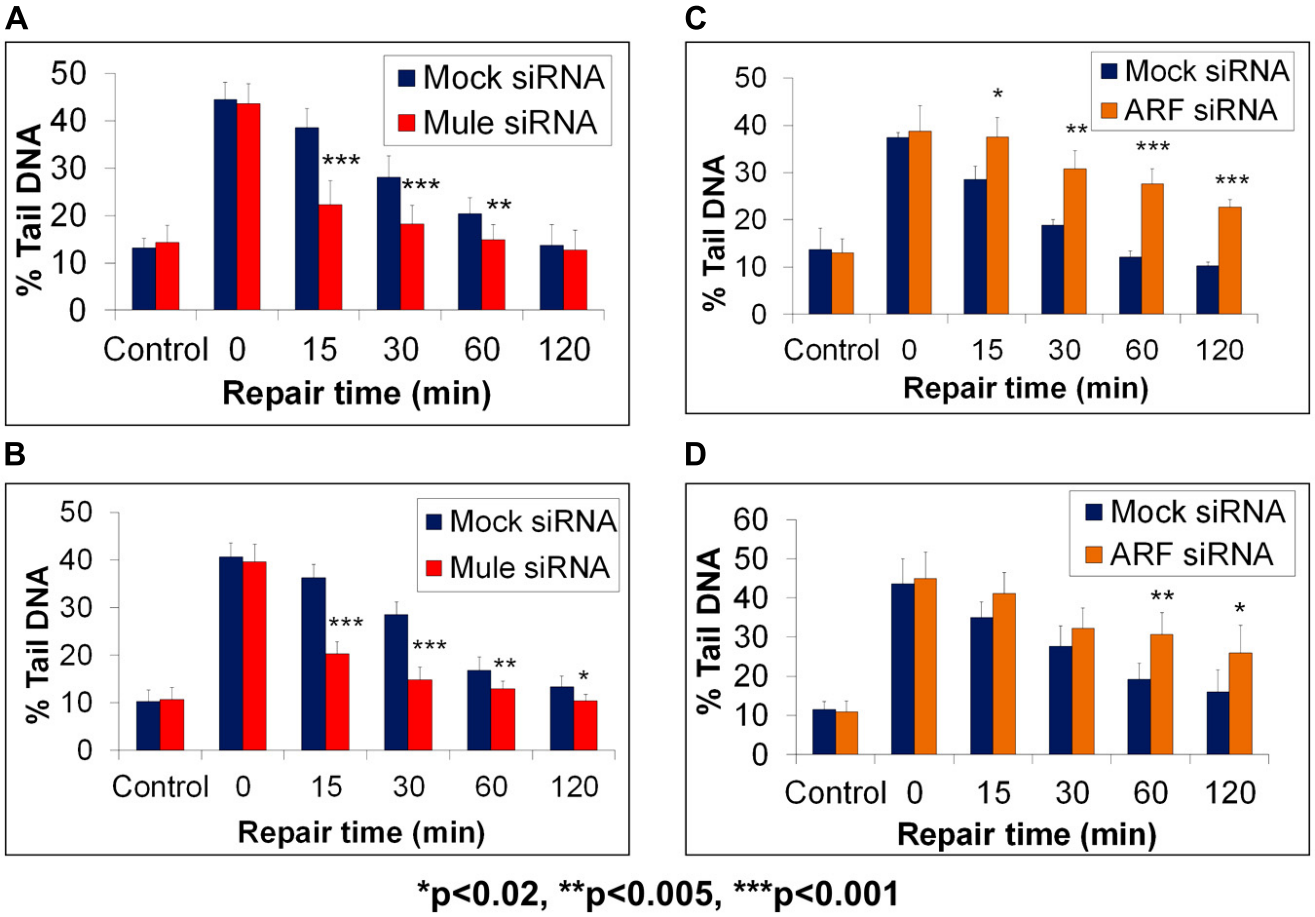
**Modulation of BER through Mule-dependent regulation of Pol β protein levels, as revealed by the in-gel DNA repair comet assay.** HeLa cells **(A,C)** or WI-38 cells **(B,D)** were treated with Lipofectamine transfection reagent (Life Technologies, Paisley, UK) in the absence (Mock siRNA) and presence of Mule siRNA **(A,B)** or ARF siRNA **(C,D)** for 72 h. Cells were analyzed using the in-gel DNA repair comet assay following treatment in suspension with 20 μM hydrogen peroxide for 5 min and allowing for DNA damage repair at 37^∘^C for up to 120 min. The mean % tail DNA values with SDs from at least three independent experiments were determined using the Komet 5.5 image analysis software (Andor Technology, Belfast, UK). Statistically significant results comparing Lipofectamine and siRNA-treated cells are represented by *p < 0.02, **p < 0.005, and ***p < 0.001, as analyzed by Student’s *t*-test. Data taken and modified from Parsons et al. (2009).

As a consequence of this study, we discovered an additional level of regulation, whereby Pol β is monoubiquitylated on the same lysine residues (41, 61, and 81) by the E3 ubiquitin ligase Mule (ARF-BP1/HectH9). This monoubiquitylation of Pol β (approximate 20% of the total protein levels) occurs in the cytoplasmic portion of the cell where Mule is predominantly located, prior to subsequent polyubiquitylation-dependent degradation by CHIP (Parsons et al., 2009). Indeed, a knockdown of Mule by siRNA in HeLa or WI-38 cells led to an increase in the cellular protein levels of Pol β, interestingly in both the cytoplasm and nucleus of the cell. Consequently, we used the in-gel DNA repair alkaline comet assay to demonstrate that as a result of increased Pol β following Mule siRNA targeted knockdown, this led to accelerated DNA damage repair rates following oxidative stress. Specifically, when HeLa (**Figure 4A**) or WI-38 cells (**Figure 4B**) were treated with hydrogen peroxide, to induce DNA damage formation, the levels of DNA strand breaks and alkali labile sites discovered by the comet assay were found to be equal in both the presence (red bars) or absence (blue bars) of Mule siRNA (see time 0). However, even at early DNA repair time points (15 min post-treatment), the levels of DNA damage were significantly reduced in the absence of Mule, compared to mock siRNA treated cells. This demonstrates more efficient DNA repair kinetics of hydrogen peroxide induced DNA damage when the Mule protein was absent. Both Mule-proficient and Mule-deficient cells were eventually able to repair all the DNA strand breaks and alkali labile sites initially induced, within the 2 h repair time period, although Mule-deficient cells were able to achieve this within a much shorter time frame (approximately 30–60 min). The observed increased DNA repair rate in cells in the absence of Mule, is consistent with the hypothesis that this is as a direct consequence of increased cellular Pol β protein levels. Since the ARF tumor suppressor protein had previously been discovered to bind and inhibit the E3 ubiquitin ligase activity of Mule (Chen et al., 2005), we conversely depleted ARF using siRNA, which we showed led to an increase in the levels of monoubiquitylated Pol β at the expense of the native protein (Parsons et al., 2009). As a consequence of an siRNA-mediated knockdown of ARF, we again were able to demonstrate using the in-gel DNA repair alkaline comet assay, that DNA repair rates of hydrogen peroxide-induced DNA damage were significantly altered in HeLa (**Figure 4C**) or WI-38 cells (**Figure 4D**). Therefore whilst mock siRNA treated cells (blue bars) demonstrated a complete reduction in the levels of DNA strand breaks and alkali labile sites visualized by the comet assay within 60–120 min post-treatment, cells in the absence of ARF (orange bars) displayed reduced DNA damage repair kinetics. In particular, significantly increased levels of DNA strand breaks and alkali labile sites were still present in ARF-depleted cells 60–120 min post-treatment. This is consistent with the observed reduced levels of Pol β in these cells that is unable to support efficient DNA damage repair rates. However, to support the hypothesis that Mule and ARF regulation of Pol β was directly involved in the modulation of the kinetics of DNA damage repair following hydrogen peroxide treatment, Mule knockdown experiments were performed in Pol β-proficient (Pol β^+/+^) and Pol β-deficient (Pol β^-/-^) cells. Therefore, in combination with the in-gel DNA repair alkaline comet assay, we revealed that the accelerated DNA repair rates of hydrogen peroxide induced DNA damage observed in HeLa and WI-38 cells following Mule siRNA, could be replicated in Pol β^+/+^ cells, as demonstrated by significantly reduced levels of DNA strand breaks and alkali labile sites at early time-points (15 and 30 min) post-treatment (Parsons et al., 2009). In contrast, DNA damage repair rates in Pol β^-/-^ cells in the absence and presence of Mule were not significantly different throughout the repair time period, suggesting the dependence of Pol β in DNA repair modulation by Mule.

**FIGURE 5:**
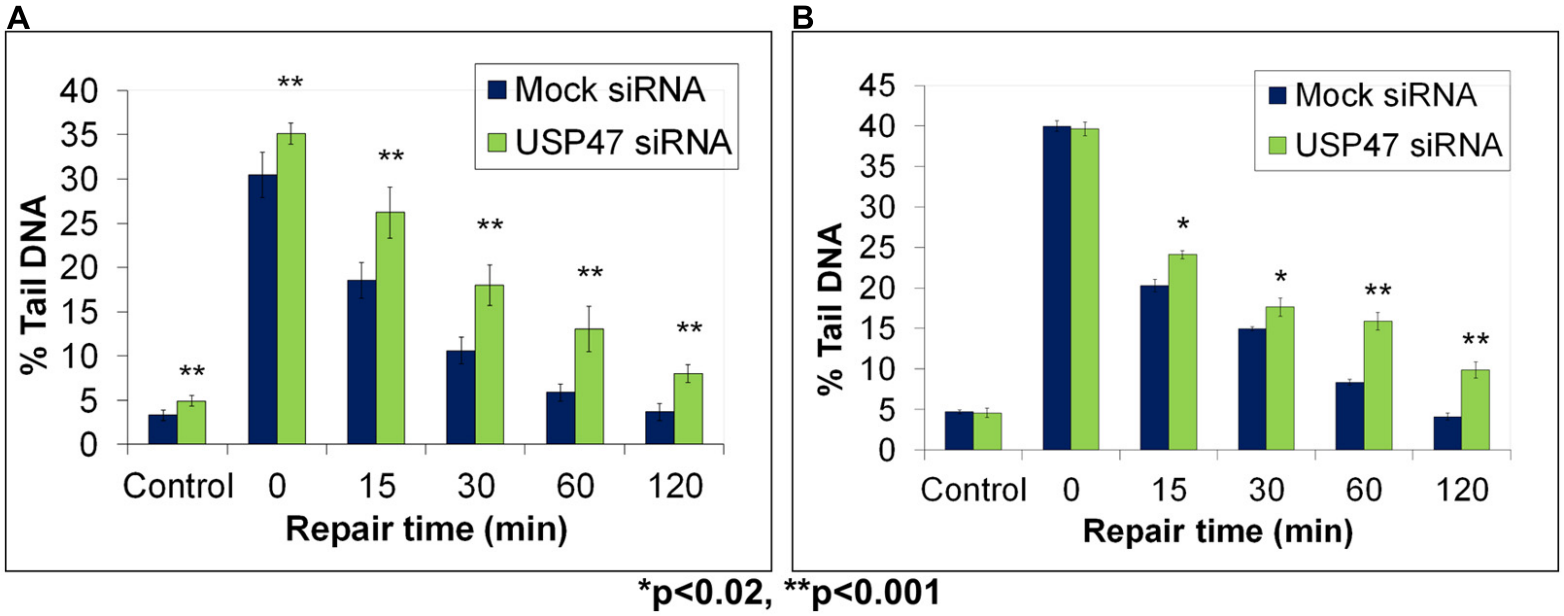
**Modulation of BER through USP47-dependent regulation of Pol β protein levels, as revealed by the in-gel DNA repair comet assay.** HeLa cells were treated in the absence (Mock siRNA) and presence of USP47 siRNA for 72 h. Cells were then analyzed using the in-gel DNA repair comet assay following treatment in suspension with **(A)** 20 μM hydrogen peroxide for 5 min or **(B)** 8 Gy ionizing radiation and allowing for DNA damage repair. Mean % tail DNA values with SDs were calculated. Further details are provided in **Figure 4** legend. Statistically significant results comparing Lipofectamine and siRNA-treated cells are represented by *p < 0.02 and **p < 0.001, as analyzed by Student’s *t*-test. Panel **(A)** taken and modified from Parsons et al. (2011) and Panel **(B)** represents unpublished data.

Whilst we had uncovered roles for the E3 ubiquitin ligases CHIP and Mule, and the ARF tumor suppressor protein in the regulation of the steady state Pol β protein levels, it was unclear how this mechanism could efficiently change in response to changes in the DNA damage environment. It was predicted that a deubiquitylation enzyme may exist that is able to rapidly reverse the effects of mono- and polyubiquitylation of Pol β, and therefore generate more active protein that is required for DNA damage repair. Similar to the studies described above, we used fractionated cell extracts but this time in combination with an *in vitro* deubiquitylation assay incorporating monoubiquitylated Pol β as a substrate, to purify and identify the major Pol β-dependent deubiquitylation enzyme (Parsons et al., 2011). This enzyme was revealed as USP47, a predominantly cytoplasmic protein. Intriguingly, we discovered that USP47 was able to deubiquitylate both mono- and polyubiquitylated forms of Pol β *in vitro*. Following a knockdown of USP47 by siRNA, we observed decreased levels of Pol β in the cytoplasmic compartment of HeLa cells, at the expense of an increase in the monoubiquitylated form of the protein. Whilst an elevation in Pol β protein levels in the nucleus following exogenous DNA damage treatment had previously been observed under normal conditions (Parsons et al., 2008), this was prevented following USP47 siRNA knockdown, suggesting that these cells may be deficient in DNA repair. Therefore, we used the in-gel DNA repair alkaline comet assay to analyze DNA damage repair kinetics of cells in the presence and absence of USP47. We demonstrated that, intriguingly, the levels of DNA strand breaks and alkali labile sites were significantly elevated in HeLa cells deficient in USP47 (**Figure 5A**; green bar) compared to mock siRNA-treated control cells (**Figure 5A**; blue bar) immediately following treatment of the cells with hydrogen peroxide (see time 0). We were also able to show that the repair of this hydrogen peroxide-induced DNA damage was defective throughout the repair time course (15–120 min) in USP47 knockdown cells compared to control cells (**Figure 5A**). In fact, significant levels of DNA damage were still observed 120 min post-treatment with hydrogen peroxide in USP47-deficient cells, highlighting reduced DNA damage repair rates. In addition to this, we now show that cells transfected with USP47 siRNA are also deficient in the repair of DNA damage induced by ionizing radiation (**Figure 5B**; unpublished data). Immediately following ionizing radiation treatment, the levels of DNA strand breaks and alkali labile sites visualized by the in-gel DNA repair alkaline comet assay were similar in Mock siRNA (blue bar) and USP47 siRNA treated cells (see time 0). In contrast following DNA repair, elevated levels of this DNA damage was specifically observed in the USP47 deficient cells at 15–120 min post-irradiation compared to control cells. Cumulatively, these data highlight the importance of USP47, and indeed the regulation of Pol β protein levels, in coordinating an efficient DNA damage repair response.

In summary, these studies have demonstrated important roles for the E3 ubiquitin ligase CHIP and Mule, the tumor suppressor ARF, and the deubiquitylation enzyme USP47 as the major enzymes of the UPP involved in controlling the steady state, and DNA damage-induced, levels of Pol β. This is achieved by controlling the stability of newly synthesized cytoplasmic Pol β, which is used as a source for nuclear Pol β required for DNA damage repair. The in-gel DNA repair alkaline comet assay employed in these studies has been instrumental in examining precise repair kinetics of DNA damage induced by oxidative stress. This method has also been key in improving our understanding of the importance of Pol β regulation in this process, by measuring DNA damage repair rates following modulation of UPP associated enzymes.

### REGULATION OF PNKP PROTEIN LEVELS

**FIGURE 6:**
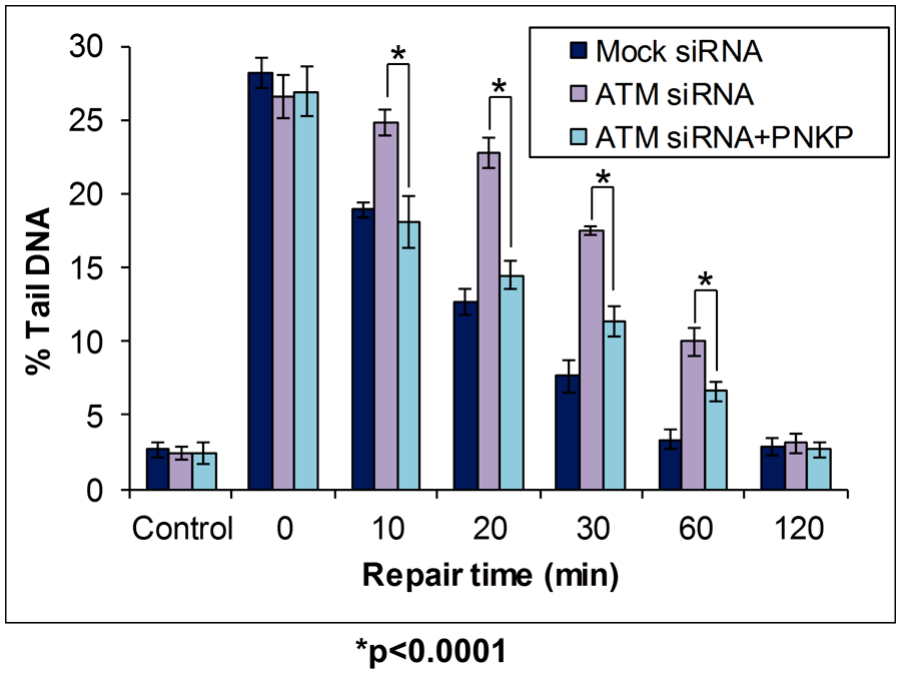
**Modulation of BER through Cul4A–DDB1–STRAP- dependent regulation of PNKP protein levels, as revealed by the in-gel DNA repair comet assay.** HCT116p53^+/+^ cells were treated in the absence or presence of ATM siRNA for 24 h. Cells were then further treated in the absence (Mock siRNA and ATM siRNA) and presence (ATM siRNA + PNKP) of a mammalian expression plasmid expressing Flag-tagged PNKP for a further 24 h. Cells were analyzed using the in-gel DNA repair comet assay following treatment in suspension with 35 μM hydrogen peroxide for 5 min, and allowing for DNA damage repair. Mean % tail DNA values with SDs were calculated. Further details are provided in **Figure 4** legend. Statistically significant results comparing ATM siRNA versus ATM siRNA-treated cells transfected with a plasmid containing Flag-tagged PNKP are represented by *p < 0.0001, as analyzed by Student’s *t*-test. Data taken and modified from Parsons et al. (2012).

BER of oxidative DNA base damage that is specifically initiated by the NEIL DNA glycosylases (NEIL1-3) generates a single nucleotide gap flanked by 5′-phosphate and 3′-phosphate DNA ends (**Figure 1**; left branch). Since the 3′-phosphate is not amenable to Pol β activity, through the insertion of the corrected undamaged nucleotide, this requires removal by the 3′-phosphatase activity of PNKP (Wiederhold et al., 2004). The importance of PNKP in the cellular DNA damage response has been demonstrated by the observation that an shRNA knockdown of PNKP caused an elevation in the sensitivity of A549 human lung adenocarcinoma cells to oxidative stress induced by hydrogen peroxide and ionizing radiation (Rasouli-Nia et al., 2004). Furthermore, reduced PNKP protein levels caused by a *pnkp* gene mutation have been found in patients suffering from a disease associated with severe neurological abnormalities, termed microcephaly, early-onset, intractable seizures and developmental delay (MCSZ), and lymphoblasts from these patients were found to be defective in the repair of oxidative DNA damage (Shen et al., 2010). These studies have therefore demonstrated the importance of regulating PNKP protein levels in the cellular response to oxidative stress, and in the prevention of human disease. We recently investigated the cellular mechanism of regulation of PNKP protein levels, and discovered that this mechanism involves a cross-talk between phosphorylation and ubiquitylation of the protein, which modifies its ability to be degraded by the proteasome. Whilst ATM-dependant phosphorylation of PNKP on serines 114 and 126 had previously been shown to occur in response to ionizing radiation by two separate studies (Segal-Raz et al., 2011; Zolner et al., 2011), PNKP phosphorylation was suggested to be required for DNA double strand break repair. However, we also discovered that this site-specific PNKP phosphorylation mediated by ATM was also induced following oxidative stress induced by hydrogen peroxide treatment in HCT116p53^+/+^ colorectal carcinoma cells (Parsons et al., 2012). This DNA damage-dependent induction in PNKP phosphorylation was associated with an accumulation of the protein (approximately 50% increase in protein levels), which was found to be mediated through inhibition of ubiquitylation-dependant proteasomal degradation of PNKP (on lysines 414, 417, and 484 within PNKP) catalyzed by the Cul4A–DDB1–STRAP E3 ubiquitin ligase complex. To demonstrate that this cellular mechanism for PNKP regulation has an impact on DNA damage repair, we used the in-gel DNA repair comet assay to measure the kinetics of repair of DNA damage induced by oxidative stress, in the presence and absence of ATM which controls cellular PNKP protein levels (**Figure 6**). Following a knockdown of ATM using siRNA in HCT116p53^+/+^ cells, we showed that these cells (purple bars) have equivalent levels of DNA strand breaks and alkali labile sites generated immediately followed hydrogen peroxide treatment (see time 0) in comparison to mock siRNA treated control cells (dark blue bars). However, following a time course of incubation of cells post-treatment, ATM knockdown cells show an elevation in the levels of DNA damage, specifically between 10 and 60 min post-treatment compared to mock-treated cells, demonstrating that ATM-depleted cells have a DNA damage repair rate defect. ATM-knockdown cells were eventually able to repair the DNA damage fully within 2 h, whereas this was achieved within 1 h in the control cells, highlighting that ATM-deficient cells are able to repair oxidative DNA damage albeit at a slower rate. Since we hypothesized that this defective DNA repair was due to the inability of ATM-deficient cells to elevate cellular PNKP protein levels in response to oxidative stress, we transfected these cells with a mammalian expression plasmid for PNKP. This leads to an elevation in the total protein levels of PNKP in these cells, which was equivalent to that observed in mock-siRNA treated cells following DNA damage induction. Consequently, we demonstrated that expression of PNKP is able to partially reverse the DNA damage repair defect seen in cells in the absence of ATM alone. Specifically, between 10 and 60 min post-treatment with hydrogen peroxide, the ATM-depleted cells complemented with PNKP (light blue bars) show less accumulation of DNA strand breaks and alkali labile sites, visualized by the in-gel DNA repair comet assay, than ATM-depleted cells alone (purple bars). However, expression of PNKP is still unable to fully correct the DNA damage repair defect, since these cells still showed increased levels of DNA strand breaks and alkali labile sites compared to mock-siRNA treated cells (dark blue bars), particularly between 20 and 60 min post-treatment. Nevertheless, this study demonstrated that ATM-dependant phosphorylation of PNKP, which is required to elevate the levels of PNKP through inhibition of ubiquitylation dependent proteasomal degradation, is required for the efficient repair of DNA damage induced by oxidative stress.

### REGULATION OF OTHER BER PROTEIN LEVELS

In addition to Pol β and PNKP, there is accumulating evidence that other BER protein levels are regulated through ubiquitylation-dependent degradation by the UPP, and therefore we will summarize some of the key important findings. APE1, the major AP endonuclease activity employed in BER, has been shown to be polyubiquitylated on lysines 24, 25, and 27 by the mouse double minute 2 (MDM2) E3 ubiquitin ligase (Busso et al., 2009), which is also the major E3 ubiquitin ligase involved in the regulation of the p53 tumor suppressor protein. It was shown by transfection of HCT116p53^+/+^ and HCT116p53^-/-^ cells with an expression plasmid for APE1, that the protein was ubiquitylated in a p53-dependant manner in the presence of DNA damage, and that increased APE1 protein was evident following MDM2 siRNA knockdown. Although more recently, UBR3 was suggested as the major E3 ubiquitin ligase purified from cell extracts that ubiquitylates APE1, within the N-terminus of the protein (Meisenberg et al., 2012). Indeed, *Ubr3*^-/-^ mouse embryonic fibroblasts displayed increased cellular levels of APE1 and were found to be genetically unstable. Both XRCC1 and DNA ligase IIIα, which are known to form a stable complex in human cells that performs the final ligation step in the short patch BER pathway, have independently been shown to be polyubiquitylated *in vitro* by the E3 ubiquitin ligase CHIP (Parsons et al., 2008). This study demonstrated that the levels of both proteins increased following CHIP depletion by siRNA in HeLa cells, demonstrating that CHIP also regulates the stability of these proteins *in vivo*. DNA glycosylases that perform the initial excision step of damaged DNA base removal are also increasingly being identified as targets for ubiquitylation-dependant proteasomal degradation. Specifically OGG1, which is the major enzyme involved in the excision of the mutagenic base 8-oxoguanine, has been shown to be a target for CHIP ubiquitylation, but only in response to hyperthermia due to the protein undergoing thermal unfolding (Fantini et al., 2013). The DNA glycosylase MutYH, that excises adenine residues opposite 8-oxoguanine that are misincorporated during DNA replication of 8-oxoguanine:cytosine base pairs, has been shown to be a target for Mule ubiquitylation both *in vitro* and *in vivo* (Dorn et al., 2014). It was observed that an siRNA knockdown of Mule in HEK293T cells caused an elevation in the cellular levels of MutYH, and that a ubiquitylation deficient mutant of MutYH was similarly more stable following transfection into HEK293T cells. It should also be noted that there is plentiful evidence highlighting an important role for modification of thymine DNA glycosylase (TDG) with the small ubiquitin modifier (SUMO; Hardeland et al., 2002; Steinacher and Schar, 2005; Smet-Nocca et al., 2011). Whilst this topic is beyond the scope of the current review, SUMOylation of TDG has been shown to regulate its DNA glycosylase activity, rather than cellular protein levels. However there is recent evidence suggesting that TDG levels are also regulated by ubiquitylation, although the E3 ubiquitin ligase catalyzing ubiquitylation-dependent degradation of TDG is currently unknown (Moriyama et al., 2014). Finally, the cellular protein levels of DNA polymerase λ which is a close relative of Pol β, has also been shown to be a target for ubiquitylation-dependant degradation initiated by Mule. DNA polymerase λ protein degradation was discovered to be inhibited by Cdk2/cyclin A-dependant phosphorylation in late S and G2 phases of the cell cycle, which promotes recruitment of DNA polymerase λ to chromatin to assist in the repair of 8-oxoguanine DNA base damage (Markkanen et al., 2012).

## CONCLUDING REMARKS

In this review, we have highlighted the increasing number of studies demonstrating a vital role for the UPP in the modulation of the cellular steady state levels of key BER proteins. These mechanisms coordinate moderate increases (∼20–50%) in BER protein levels in the cellular response to DNA damage. This mechanism of BER protein regulation is ultimately performed by substrate specific E3 ubiquitin ligases and deubiquitylation enzymes that either add ubiquitin moieties onto target proteins, or conversely remove them, and therefore modulate their degradation by the 26S proteasome. Interestingly, misregulation of BER proteins is frequently observed in several human disorders, such as in aging, cancer, and neurodegenerative diseases. Therefore the next goal will be to examine the role of the UPP, and the enzymes therein, in this disease-dependent misregulation that may reveal the mechanistic processes involved. Particularly in the case of human cancer, this research may also uncover novel cellular targets for drugs or small molecule inhibitors, which when combined with radiotherapy and/or chemotherapy, may generate novel therapeutic strategies for curing the disease. In this review, we have also described our use of a modified comet assay, where DNA repair activities are monitored by allowing cultured cells to repair in-gel prior to cell lysis and DNA electrophoresis. This method has allowed us to monitor changes in BER regulation via modulation of enzymes involved in the UPP, and has been key to demonstrating the effect of this process in coordinating an efficient cellular response to DNA damage. The modified comet assay has also enabled us to avoid the extensive cell culture-based processing associated with the more traditional approach using attached cells, as discussed earlier. This technique allows for the determination of much more precise DNA repair kinetics at various time points post-treatment (i.e., with hydrogen peroxide or ionizing radiation) and is our preferred method for measuring the repair of DNA strand breaks and alkali-labile sites in the various cell lines that we routinely use.

## Conflict of Interest Statement

The authors declare that the research was conducted in the absence of any commercial or financial relationships that could be construed as a potential conflict of interest.
